# Miller–Fisher syndrome after first dose of Oxford/AstraZeneca coronavirus disease 2019 vaccine: a case report

**DOI:** 10.1186/s13256-022-03592-4

**Published:** 2022-11-16

**Authors:** Fernanda Junqueira Cesar Pirola, Bruno Antônio Müzel Santos, Gabriela Feres Sapienza, Lucas Yuri Cetrangolo, Caio Henrique Wthen Gambacorta Geranutti, Paulo Henrique Pires de Aguiar

**Affiliations:** 1 Department of Medical Science, Medical School, Catholic Pontifical University of Sao Paulo, Sorocaba, Brazil; 2 Department of Neurology, Division of Clinical Medicine, Catholic Pontifical University of Sao Paulo, Sorocaba, Brazil; 3 Department of Neurology, Division of Neurology, Catholic Pontifical University of Sao Paulo, Sorocaba, Brazil

**Keywords:** Miller Fisher syndrome, Guillain–Barré syndrome, COVID-19, COVID-19 vaccine, Oxford vaccine, AstraZeneca vaccine, Ophthalmoplegia, Areflexia, Ataxia

## Abstract

**Introduction:**

Miller-Fisher Syndrome (MFS) is a variant of Guillain–Barré syndrome (GBS), an acute immune-mediated neuropathy, which manifests as a rapidly evolving areflex motor paralysis. This syndrome presents as a classic triad: ophthalmoplegia, areflexia, and ataxia. MFS is usually benign and self-limited.

**Case report:**

A Caucasian patient was admitted to our hospital with the flu, loss of bilateral strength in the lower limbs and upper limbs and sudden-onset ataxia 7 days after receiving a first dose of the Oxford/AstraZeneca COVID-19 vaccine. On neurological examination, the patient had Glasgow Coma Scale score of 15, with absence of meningeal signs; negative Babinski sign; grade 2 strength in the lower limbs and grade 4 strength in the upper limbs; axial and appendicular cerebellar ataxia; and peripheral facial diparesis predominantly on the right, without conjugate gaze deviation. Cerebrospinal fluid (CSF) was collected on admission, and analysis revealed albuminocytological dissociation with CSF protein of 148.9 mg/dL; leukocytes, 1; chlorine, 122; glucose, 65 mg/mL; red cells, 2; and non-reactive venereal disease research laboratory test result. The COVID-19 IgG/IgM rapid immunological test was negative. Electroneuromyography revealed a recent moderate-grade and primarily sensory and motor demyelinating polyneuropathy with associated proximal motor block.

**Discussion and conclusion:**

Miller-Fisher Syndrome may be related to events other than infections prior to neuropathy, as in the case reported here. The patient presented strong correlations with findings for MFS reported in the literature, such as the clinical condition, the results of electroneuromyography, and results of the CSF analysis typical for MFS. When treatment was provided as proposed in the literature, the disease evolved with improvement. Ultimately, the diagnosis of incomplete MFS was made, including acute ataxic neuropathy (without ophthalmoplegia).

## Introduction

Guillain–Barré syndrome (GBS) is currently recognized as a group of acute immune-mediated neuropathies and the most common and severe acute paralytic neuropathy worldwide, with about 100,000 people developing the disease each year [1, 2].

The incidence rates of GBS in Europe and North America show a range of 0.8–1.9 cases per 100,000 people per year. This rate increases with age (0.6 per 100,000 per year in children and 2.7 per 100,000 per year in the elderly aged ≥ 80 years). It is more frequent in men than in women and, in Western countries, adults are affected more frequently than children [1, 3]. The incidence of GBS is higher in winter than in summer in some areas of the world, possibly associated to the prodromal periods of some infectious agents [4].

GBS manifests as a rapidly evolving areflexic motor paralysis, with or without sensory changes. The usual pattern is a rapidly evolving ascending paralysis. It may accompany dysesthesia, with tingling in the limbs and areflexia or hyporeflexia. In addition, pain in the neck, shoulders, back, or diffusely in the spine is common in the early stages of GBS, occurring in about 50% of patients. The lower limbs are usually more affected than the upper limbs and facial diparesis is present in 50% of the affected individuals. Inferior cranial nerves can be affected and bulbar weakness may also occur, affecting muscles involved in breathing, swallowing, speech, and tongue movement [3, 5]. Clinical worsening continues for 4 weeks, but thereafter further progression is unlikely. Up to 90% of patients with GBS have maximal weakness during this period [3, 5].

There is a variety of GBS syndromes that are limited or regional and that have axonal or demyelinating characteristics. An example is Miller–Fisher syndrome (MFS), which presents with the classic triad: ophthalmoplegia, areflexia and ataxia. MFS accounts for about 5% of all cases of GBS, with an incidence of 1–2 cases per 1,000,000 people, and is strongly associated with antibodies against the ganglioside GQ1b. 3.4 [3, 4].

Due to MFS being a variant of GBS, it is of great importance to highlight the numerous case reports and studies that have linked GBS to coronavirus disease 2019 (COVID-19) caused by severe acute respiratory syndrome coronavirus 2 (SARS-CoV-2), even though no definitive causal association of GBS with the use of vaccines has been proven [6–12].

## Case description

### Anamnesis

A 47-year-old Caucasian woman (APS) was admitted by the neurology team with flu, loss of strength bilaterally in the lower limbs and upper limbs, and sudden onset ataxia 7 days after receiving Oxford/AstraZeneca COVID-19 vaccine. After 3 days of hospitalization, she progressed with partial improvement in the upper limbs and worsening loss of strength in the lower limbs, causing her to be bedridden. In addition, she started to present with axial cerebellar ataxia, dysphonia.

### Physical examination

Clinical examination revealed a woman with regular general health status, no fever, acyanotic, and anicteric. Neurological examination revealed: Glasgow Coma Scale score of 15, no meningeal signs; negative Babinski sign; strength grade 2 in the lower limbs and strength grade 4 in the upper limbs; axial and pendicular cerebellar ataxia; and peripheral facial diparesis predominantly on the right, without conjugate gaze deviation.

### Laboratory tests

Cerebral spinal fluid (CSF) was collected upon admission and tested in the laboratory. The results showed albuminocytological dissociation, with protein, 148.9; leukocytes, 1; chlorine, 122; glucose, 65 mg/mL; red blood cells, 2; and non-reactive venereal disease research laboratory test result. The hematological test results were: hemoglobin, 14.8 g/dL; hematocrit, 42.9%; leukocytes, 10.8 mL/mm^3^ with no left shift; platelets, 274 × 10^3^/mm^3^; potassium, 4.1 mEq/L; sodium, 138 mEq/L; calcium, 1.118 mEq/L; urea, 38 mg/dL; creatinine, 0.8 mg/dL; and C-reactive protein (CRP), 5.6 mg/L.

A number of other tests were also performed following admission, including: lactic dehydrogenase, 290 U/L (normal range: 125–220 U/L); gamma glutamyl transferase (GGT), 56 U/L (normal range: 9–36 U/L); PCR test, 10.8 mg/L. The patient tested negative for the COVID-19 IgG/IgM rapid immunological test.

Electroneuromyography (ENMG) revealed a recent and moderate primarily motor and sensory demyelinating polyneuropathy with proximal motor block. The ENMG report was correlated with the patient’s clinical condition to consider acute polyradiculoneuritis, which led to the hypothesis of MFSwhen associated with a cerebellar ataxic component.

### Imaging exams

One week before admission to the hospital, the head computed tomography (CT) showed mineral deposition in the basal ganglia. Head CT was normal on the day of admission.

### Management and evolution

Intravenous immunoglobulin (IVG) was administered, 0.4 g/kg/day for 5 days, associated with motor physiotherapy, which led to improvement of the neurological symptoms during hospitalization.

### Prognosis and follow-up

The patient was discharged with follow-up of motor physical therapy and use of gabapentin 300 mg every 12 hours for pain control. She was advised to seek emergency care if neurological condition worsened. At this point, plasmapheresis may be considered.

## Discussion

Guillain–Barré Syndrome is a monophasic immune-mediated disease, usually triggered by a previous infection [13]. Within its spectrum, 30–50% of patients with MFS have had previous infections that could be identified as a possible trigger for an immune-mediated process involving peripheral nerves and nerve roots [14]. Similar to GBS, MFS is often associated with a previous antigenic stimulus, such as an infectious disease. The most common association is with recent infection by *Campylobacter jejuni* [15]. Other less common events or associated diseases includes cytomegalovirus, influenzae, *Mycoplasma pneumoniae*, the flaviviruses, including the Zika and dengue viruses, and the alpha virus chikungunya [6]. However, some studies have described the occurrence of GBS and MFS as being related to other events, one of which is vaccination [7].

MFS is considered a benign, self-limited disease, and the prognosis is generally quite favorable, with most patients showing complete resolution of signs and symptoms within 6 months [2, 15]. In practice, it is often accompanied by cranial nerve involvement and may progress to limb weakness (Miller Fisher-Guillain–Barré overlap syndrome). Likewise, MFS may present only as an isolated ocular nerve paralysis [1]. The fact that the patient did not have ophthalmoplegia does not exclude the hypothesis of MFS as it fits the incomplete form of acute ataxic neuropathy.

Also, among the variants of GBS, MFS presents with axonal or demyelinating characteristics, which is an important fact considering that the ENMG showed a sensory and motor demyelinating polyneuropathy with proximal motor block [3, 4].

A lumbar puncture and CSF analysis of patients with GBS usually helps diagnosising the syndrome by revealing albuminocytological dissociation, which shows an increase in CSF protein level but a normal cell count. This finding was evident in the CSF results of the present case and is initially present in about 50-66% of patients with GBS after the first week of symptom presentation [13, 16].

As mentioned, vaccination can be a trigger for MFS, an important fact in the context of the COVID-19 pandemic and consequent development of new vaccines to combat the virus. Several side effects have been reported in clinical trials following the use of different types of vaccines present on the market today [17, 18]. However, the occurrence of GBS after COVID-19 vaccination has been reported only a few times to date [6, 19–21].

In the case reported, the patient presented symptoms after receiving the first dose of the AstraZeneca vaccine. In this context, the first case of GBS in the literature occurred after the person received an initial dose of the Pfizer-BioNTech COVID-19 vaccine [21]. In another documented case, this one in a person who received the Oxford-AstraZeneca SARS-CoV-2 vaccine, surprisingly similar clinical features were reported, with symptoms presented after the first dose, in addition to ataxia, ophthalmoplegia and areflexia. The two cases support the possibility of a similar pathogenic mechanism [22].

There have also been reports of GBS occurring within 11–22 days of vaccination with the Oxford-AstraZeneca SARS-CoV-2 vaccine. However, it must be noted that it is possible to correlate the onset of these symptoms with the period in which the maximum immune response to the vaccine occurs [19]. COVID-19 vaccination generally has a favorable prognosis, with remarkable recovery within a few weeks, as reported in previous studies [22, 23].

The pathogenesis of GBS and MFS after vaccination with COVID-19 is not fully described in the literature. However, it probably occurs due to an abnormal immune-mediated response. One of the hypotheses of the proposed pathogenic mechanism is that of molecular mimetism, where an antigen stimulates an abnormal immune response involving gangliosides on the peripheral nerves [23].

Clinical trials using Oxford-AstraZeneca reported a prevention rate of 70% of COVID-19 cases [24]. Therefore, the literature indicates that the benefits of the vaccine outweigh its side effects [2, 6].

In summary, acute ataxic neuropathy (without ophthalmoplegia) was indicated as the best diagnostic hypothesis since the patient presented ataxia, limb weakness, and Bell’s paralysis, an important fact since the seventh cranial nerve is the most affected by MFS. Possible involvement of other cranial nerves, such as pair X, was taken into account due to the appearance of dysphonia. In addition, because the patient presented strong correlations with the findings in the literature, such as a typical clinical scenario, typical findings from ENMG and CSF analysis, and an adequate response to the treatment, the main diagnosis was MFS. Despite being a less prevalent variant, its presentation as part of atypical GBS led the case [3, 5, 15].

## Conclusion

Miller-Fisher Syndrome is a rare complication after vaccination against COVID-19. However, it is also important that a correct diagnosis is made when a patient presents with associated signs and symptoms in order to initiate early treatment. Given the still current COVID-19 scenario, it is of great importance that new studies describe and detail the correlation between COVID-19 vaccination and GBS, as these will contribute to increasingly better outcomes in the medical field.

## Data Availability

Not applicable
